# Complement Activation in Nephrotic Glomerular Diseases

**DOI:** 10.3390/biomedicines12020455

**Published:** 2024-02-18

**Authors:** Dominik Nell, Robert Wolf, Przemyslaw Marek Podgorny, Tobias Kuschnereit, Rieke Kuschnereit, Thomas Dabers, Sylvia Stracke, Tilman Schmidt

**Affiliations:** Section of Nephrology, Clinic and Policlinic of Internal Medicine A, University Medicine Greifswald, Ferdinand-Sauerbruch-Straße, 17475 Greifswald, Germany; dominik.nell@stud.uni-greifswald.de (D.N.);

**Keywords:** nephrotic syndrome, podocyte, complement, glomerular diseases, membranous nephropathy, membranoproliferative glomerulonephritis, lupus nephritis, focal segmental glomerulosclerosis, minimal change disease

## Abstract

The nephrotic syndrome holds significant clinical importance and is characterized by a substantial protein loss in the urine. Damage to the glomerular basement membrane or podocytes frequently underlies renal protein loss. There is an increasing belief in the involvement of the complement system, a part of the innate immune system, in these conditions. Understanding the interactions between the complement system and glomerular structures continually evolves, challenging the traditional view of the blood–urine barrier as a passive filter. Clinical studies suggest that a precise inhibition of the complement system at various points may soon become feasible. However, a thorough understanding of current knowledge is imperative for planning future therapies in nephrotic glomerular diseases such as membranous glomerulopathy, membranoproliferative glomerulonephritis, lupus nephritis, focal segmental glomerulosclerosis, and minimal change disease. This review provides an overview of the complement system, its interactions with glomerular structures, and insights into specific glomerular diseases exhibiting a nephrotic course. Additionally, we explore new diagnostic tools and future therapeutic approaches.

## 1. Introduction

Nephrotic syndrome (NS) is a glomerular disease of clinical significance and is characterized by proteinuria, hypoalbuminemia, edema, and dyslipidemia [1]. Under physiological conditions, the unique structure of the glomeruli ensures the control of glomerular capillary pressure and a stable glomerular filtration rate (GFR). The glomerular filtration barrier (GFB), comprising specialized endothelial cells, the glomerular basement membrane (GBM), and the podocytes, allows for the filtration of water and small solutes, such as sodium and urea. At the same time, it prevents the passing of large molecules or cellular blood components [2]. Healthy individuals typically exhibit only small quantities of detectable urinary albumin, and even proteins with low molecular weights undergo complete reabsorption in the proximal tubules.

However, nephrotic syndrome is characterized by massive proteinuria, usually exceeding 3500 mg daily. Proteinuria occurs due to GFB dysfunction, typically localized within the GBM or podocytes. While albumin also reaches the primary urine under physiological conditions, the reabsorption mechanisms of proximal tubular cells become insufficient in the presence of a GFB dysfunction, resulting in albuminuria and tubular damage. The loss of albumin through the GFB and impaired tubular reabsorption lead to hypoalbuminemia. Additionally, there is an increased capillary permeability, resulting in the escape of albumin into the interstitial space, further driving hypoalbuminemia [1]. The clinical presentation of NS also includes the development of edema. The assumption that the loss of albumin leads to a loss of oncotic pressure and subsequent edema has mainly been discarded. Instead, there is an increased reabsorption of NaCl. Due to the presence of proteases, which end up in the urine due to proteinuria, there appears to be a proteolytic activation of the epithelial sodium channel (ENaC). Ultimately, this leads to an expansion of plasma volume and the development of edema [3,4]. The tubular resistance to atrial natriuretic peptide might further amplify the plasma volume [5]. Increased hepatic synthesis of lipoproteins and enzymes and the urinary loss of liporegulators lead to hypercholesterolemia and hypertriglyceridemia [1]. In addition, a defect of anticoagulant proteins, an increase in procoagulant factors, platelet hyperreactivity, impaired fibrinolysis, increased plasma viscosity, and urinary loss of plasminogen activator inhibitor-1 lead to significantly increased risk of thrombosis, which makes nephrotic syndrome clinically relevant [6]. Although a decrease in GFR is not necessarily the main symptom of nephrotic syndrome, the development of chronic kidney disease (CKD) is a relevant problem.

The GFB is no longer considered a simple anatomical barrier. Instead, it is now recognized as a site of signaling pathways. It is involved in the immunological diseases of the glomeruli and, for example, interact with the adaptive and innate immune system [7,8,9]. Studies further indicate that the GFB also interacts with the complement system [10,11,12]. The complement system is part of the innate immune system, consisting of numerous proteins that interact with each other, with other local cells, and immune cells. In numerous glomerulonephritides, the complement system is activated by different mechanisms [13]. Based on current clinical studies, new complement system inhibitors are expected to be approved soon.

In this review, we provide an overview of the currently known complement-mediated pathomechanisms that can lead to damage to the basement membrane and podocytes. In detail, we summarize the current knowledge on complement activation in membranous glomerulopathy, membranoproliferative glomerulonephritis, lupus nephritis, focal segmental glomerulosclerosis, and minimal change disease. We also discuss the current state of research and provide an outlook on future research findings and treatment options.

## 2. Complement

**Figure 1:**
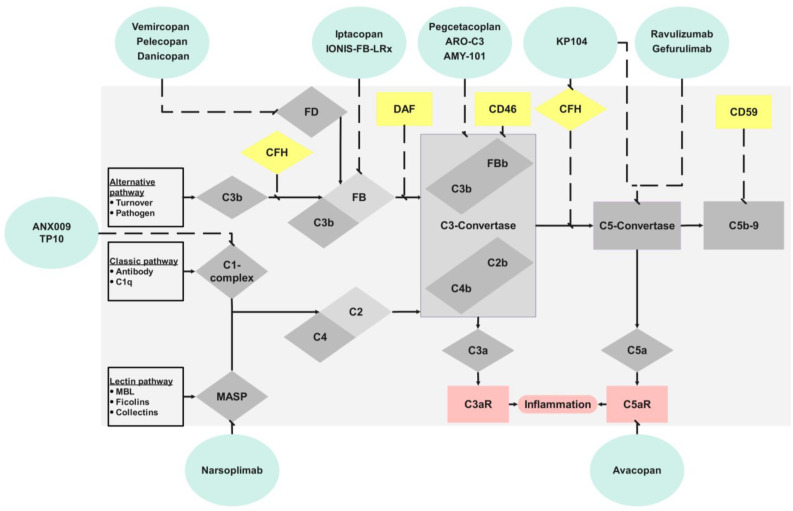
Activation (gray) and effects (pink) of the complement system through the three pathways (alternative, lectin, classical) with regulatory factors (yellow) and therapeutic complement inhibitors (light green).

The complement system consists of more than 100 soluble and membrane-bound proteins, activating and deactivating each other. Although the complement system is a part of the innate immune system, it interacts with the adaptive immune system and blood coagulation [14,15,16]. During activation of the complement system, cleavage of the essential complement factor 3 (C3) by a C3 convertase occurs. Here, as at other sites of complement activation, complement cleavage products, which have independent functions, are formed. For example, C3a and C3d mediate pro-inflammatory effects through their action as anaphylotoxins and of binding to leukocytes [17]. An important cleavage product of C3 is C3b, as it is directly involved in forming another enzyme, the C5 convertase. Cleavage of complement factor 5 (C5) produces C5b, which initiates the so-called Membrane Attack Complex (MAC; C5b-9) by attaching complement factors 6, 7, 8, and 9. The MAC ultimately forms pores in lipid bilayers, leading to the lysis of pathogens by a “multi-hit” mechanism [18]. Several studies have elucidated new functions of the MAC beyond its “classical” cytolytic (pore-forming) function. Cells exposed to sublytic MAC can instigate intracellular Ca^2+^-dependent or Ca^2+^-independent signaling pathways that affect cell proliferation, induction of apoptosis, cell motility, inflammasome activation, and pro-inflammatory cytokine signaling [19]. At the same time, C5a, another cleavage product of C5, again represents a pro-inflammatory molecule [17]. Even though these processes are uniform, the initiation of complement activation varies (Figure 1).

In principle, there are three different pathways of complement activation: the classical pathway, the alternative pathway, and the lectin pathway. The easiest to understand is activation via antibodies, particularly by immunoglobulin G and M, which correspond to the classical activation pathway. C1q binds to antibodies in classical activation and conforms to an activated protease [19]. Cleavage of C4 and C2 forms the C3 convertase of the classical pathway (C4bC2b). In addition, pattern recognition molecules, such as mannose-binding lectin (MBL), ficolins, and collectins, mediate the cleavage of C2 and C4. Such activation occurs physiologically on bacterial surfaces and corresponds to the lectin pathway [20]. As in classical activation, the C3 convertase of the lectin pathway forms of complement factors 4 and 2 (C4bC2b). The C3 convertase then forms the origin of the common terminal pathway. As previously indicated, the generation of the complement factor C3b occurs concomitantly with the cleavage of C3. Together with cleaved complement factor B (Bb), complement activation is amplified in that C3b and Bb themselves act as C3 convertase of the alternative complement pathway (C3bBb) [21]. In addition, hydrolytic cleavage of C3b from C3 occurs continuously, so the alternative complement pathway is always activated [22].

This system necessitates regulatory mechanisms to counteract persistent activation, ensuring homeostasis between excessive and insufficient activation and adapting to the respective situation [23]. A critical regulator here is complement Factor H (CFH). CFH is a soluble protein that regulates the alternative C3 convertase and the local inhibition of complement in the glomeruli [24]. Accordingly, genetic and functional alterations of CFH were described in both atypical hemolytic uremic syndrome (aHUS) and C3 glomerulopathy (C3G) [25,26]. Regulators for the classical pathway (C4-binding protein (C4bp)) and the lectin pathway (MAP-1) also exist [27,28]. In addition to these soluble inhibitors, locally expressed complement inhibitors, such as CD46, CD59, or CD55 (DAF), are described [29,30,31].

Given that kidney cells can express complement factors themselves, this raises the question of whether the cellular structures of the kidney interact with the complement system [32].

## 3. Renal Complement Activation

Various authors postulate a role for the complement system in numerous glomerular diseases [13]. In particular, complement activation is crucial in atypical hemolytic uremic syndrome (aHUS). An increased activation of the alternative and the terminal complement pathway in this disease leads to endothelial damage. Clinically, aHUS usually presents as a thrombotic microangiopathy and acute renal failure [33].

In nephrotic syndrome, the primary damage site is the GBM, the podocyte, or both, so involvement of the complement system in glomerular injury must have other potential pathomechanisms. The GBM, produced by endothelial cells and podocytes, comprises α345(IV)collagen, laminin, nidogens, and heparan sulfate proteoglycans. The podocyte is a morphologically highly complex cell. Podocytes branch rapidly with numerous foot processes. The so-called slit diaphragm forms between the foot processes, which is a cell–cell junction harboring essential proteins such as nephrin, podocin, or synaptopodin. With the help of integrins, the foot processes also connect to the GBM [34,35,36]. The podocyte thus prevents the passage of large molecules across the GFB more than any other structure [37]. Due to its high specialization, the podocyte is susceptible to stressors, and complex biological changes occur in the podocyte, including loss of integrity and changes in cellular metabolism. Effacement of the foot processes is the final route of stressed podocytes.

However, the podocyte is no longer recognized as a simple barrier that prevents the passage of proteins into the urine. Instead, there is increasing evidence that the podocyte actively generates and regulates immune-mediated glomerular diseases, for example, by expressing Toll-like receptors and releasing chemokines and cytokines [38,39,40]. The podocyte thus actively participates in shaping a pro-inflammatory environment. The podocyte also appears to influence the course of glomerular diseases by interacting with immune cells. Podocyte expression of MHC molecules and antigen presentation with consecutive podocyte damage have already been demonstrated [8,41]. On the other hand, podocytes also appear to be able to protect themselves against immunological stress [42].

The glomerular activation of complement also represents an immunological stressor on the podocytes. However, only a strong activation of C5b-9 leads to the lysis of the podocytes. The binding of C5b-9 in sublytic doses already induces changes within the metabolism of the podocytes. The activation of the complement system on the podocyte thus potentially leads to an influence on kinases, lipid metabolism, stress on the endoplasmic reticulum, and changes in the ubiquitinin–proteasome system [43,44,45,46]. In contrast, the podocyte appears to be able to protect itself from sublytic doses of MAC. In vitro data suggest that autophagy protects the podocyte from such attacks. Lv et al. reported increased changes in disease injury-related morphology when autophagy was inhibited with 3-methyladenine, while treatment with rapamycin resulted in better podocyte survival [47]. The importance of autophagy and lysosomal degradation was confirmed in a further study [48]. In vitro studies with podocytes from patients with membranous glomerulopathy showed that although there was an increase in the number of autophagosomes in the podocytes of the patients, there was no increase in lysosomal degradation. The authors conclude from this that C5b-9 inhibits degradation in general. By examining the expression profile of complement in podocytes, it became clear that numerous complement factors can be detected using rtPCR [8]. The authors found the expression of complement factors and their receptors. After the role of pro-inflammatory complement cleavage products (e.g., C5a) had become clear, Abe et al. investigated the expression of the C5a receptor (C5aR) in various human glomerulonephritides. Using immunohistochemistry and in situ hybridization, a significant increase in C5aR was observed in the cases of IgA nephropathy and membranous glomerulopathy, including podocytes [49]. Surgically resected kidneys and minimal change disease served as controls, where the C5aR was predominantly detected tubularly by immunohistochemistry. An elegant paper elucidated the connection between complement activation on podocytes and nephrotic syndrome. Angeletti et al. investigated the role of the decay acceleration factor (DAF/CD55), a regulator of C3 convertase. The influence of DAF on podocytes was demonstrated in various mouse models. The authors assume an interaction of C3a with the podocyte, which indirectly prevents the regulation of C3 convertase [31]. Zoshima et al. found complement Factor H expressed in podocytes [10]. This work provided evidence of the functional significance of CFH production from podocytes. Using a podocyte-specific mouse model, the authors showed that increased expression of CFH is associated with improved clearance of subendothelial deposited immunoglobulin G. The mechanism by which CFH is involved in removing immunoglobulins could not be clarified in this study. The podocyte upregulates the production of VEGF under different conditions. An increased production of vascular endothelial growth factor (VEGF) also leads to an increased production of CFH. This observation was made not only in podocytes, but also in retinal cells. Blocking VEGF increased the deposition of complement factors of the alternative pathway [50]. In addition to the production of CFH, the podocyte might also be an activator of complement. In vitro data suggest the production of C3 and the cleavage into the pro-inflammatory cleavage product C3a by the podocyte [11].

Even the basement membrane regulates complement activation due to its composition. Heparan sulfate proteoglycans (HSPGs), an essential component of the GBM, appear to play a role here. It has long been assumed that HSPGs are involved in the charge selectivity of GFB. Accordingly, a loss of HSPGs has been observed in several nephrotic diseases (lupus nephritis, membranous glomerulopathy, minimal change disease). However, CFH also binds to HSPGs of the GBM. In this way, the GBM protects itself from attacks by the complement system [12].

A further potential mechanism of injury is the renal excretion of proteins and proteases in urine, which include complement factors. Urinary activation products of the complement system (C5b-9) correlate with the progression of diabetic nephropathy. Additionally, more severe tubular damage has been observed in patients with high activation products in urine [51]. In a study by Woern et al., the presence of complement factors in patients with various nephrotic conditions (including membranous nephropathy, minimal change glomerulopathy/focal segmental glomerulosclerosis, and immune complex glomerulonephritis) was demonstrated in urine. Furthermore, an increased activity of proteases from the clotting cascade and the complement cascade was observed. Examples of the latter include complement factors B and D. Therefore, a change in the composition of the complement system due to proteinuria is conceivable. However, speculation also arises about tubular kidney damage through the increased activity of proteases, leading to the activation of the complement system in urine [52].

Recently, Medica et al. published a new concept of complement regulation that goes beyond the known mechanisms. The authors showed in an in vitro model that endothelial progenitor cells could also achieve local complement regulation. These cells were able to influence endothelial cells and podocytes via vesicle-transported RNA. In a pro-inflammatory environment and in the presence of C5a, transferred RNA could maintain the functions of endothelial cells and podocytes [53].

The diverse mechanisms of activation and regulation of the complement system make a therapeutic approach in various nephrotic diseases interesting. However, a more precise understanding of the different nephrotic glomerular diseases is necessary for planning future therapies.

## 4. Nephrotic Glomerular Diseases

### 4.1. Membranous Nephropathy (MN)

MN is the most common nephrotic glomerulonephritis in adults [54]. Specific autoantibodies against podocyte-expressed phospholipase A2 receptor (PLA2R) and thrombospondin type 1 domain-containing 7 A (THSD7A) can explain about 75% of cases [55,56]. Renal biopsies are characterized by depositions of subepithelial IgG and C3, suggesting an activation of the classical complement pathway. Autoantibodies against PLA2R and THSD7A are predominantly of the IgG4 isotype. This isotype has the lowest binding capacity for the complement, so complement activation via the classical pathway is unlikely to be the primary mechanism [57]. Nevertheless, C1q and C4d deposits in the vast majority of MN support the assumption of classical activation [58,59]. On the other hand, other authors have also described the glomerular deposition of MBL in the MN. Since activation of the lectin pathway can also lead to deposits of C4d, one can interpret such findings as the primary activation of the lectin pathway [59]. However, correlations between MBL in the sera of patients and the clinical course were not meaningful. In contrast, correlations between the autoantibody titers and the clinical course are possible [60]. In addition, Bally et al. described cases of MN in patients with a genetic MBL deficiency [61]. There is also discussion about alternative complement activation in MN. Seifert et al. recently addressed the question of primary complement activation [62]. In human kidney biopsies, the authors detected deposits of C1q. As in other studies, IgG4 was the dominant isotype antibody deposition. However, Seifert et al. showed that in addition to IgG4, at least one other IgG isotype that can activate complement is deposited in the kidney. In addition, a proximity ligation assay could demonstrate a dominance of classical activation. The dominance of the classical complement activation contrasts with a study by Manral et al. Based on in vitro studies, an alternative pathway activation is primarily assumed here. In this study, the authors analyzed patients with a THSD7A-associated MN and hypothesized an alternative complement pathway activation by binding to IgG4. The observation that the binding of C3b was completely absent in factor B-depleted sera supports the assumption of an alternative activation pathway [63].

### 4.2. Membranoproliferative Glomerulonephritis (MPGN)

MPGN is a morphological pattern of glomerular changes characterized by a thickening of the glomerular basement membrane. The thickening of the basement membrane is caused by deposits of complement factors and, in some cases, immunoglobulins. MPGN can occur in numerous diseases [64]. Depending on the location of the deposits, MPGN type I shows subendothelial and mesangial deposits, type II MPGN intramembranous deposits, and type III MPGN additional subepithelial deposits. Since the composition and the localization of deposits vary, the clinical course of MPGN is very heterogeneous. A nephrotic syndrome occurs in 30–50% of cases [64,65]. The Consensus Report 2013 advocated restructuring the classification of MPGN. The objective was to pivot toward categorization based on immunohistochemical and immunofluorescence characteristics, departing from solely morphological distinctions [66,67]. A novel classification termed C3 glomerulopathy (C3G) was proposed for instances where C3 deposition surpassed that of immunoglobulins. This redefinition of C3G encompassed the patterns noted in MPGN types I and III, along with intramembranous glomerulonephritis/dense deposit disease (MPGN type II). Furthermore, the scope of diagnosing C3G was expanded beyond membranoproliferative patterns to encompass other manifestations of glomerulonephritis, such as mesangioproliferative patterns. In scenarios where immunoglobulin deposition was predominant, the nomenclature transitioned to immune complex-mediated MPGN (IC-MPGN). The evolution of this revised definition stemmed from advancements in comprehending complement-mediated kidney diseases, with C3 glomerulopathy (C3G) serving as a prototype [68]. Within C3G, an excessive activation of the complement system has been associated with genetic mutations in various complement genes, notably Factor H, C3, and the genes encoding FHR1, FHR2, FHR3, FHR4, and FHR5. A subclass of antibodies known as nephritic factors has been identified, playing a role in stabilizing complement activation by binding to elements such as the alternative C3 convertase, C5 convertase, Factor H, C3, C3b, C3d, or Factor B. These antibodies disrupt the alternative pathway, resulting in its hyperactivation. Numerous additional mutations, predominantly affecting the alternative complement pathway, have also been identified. These mutations involve genes associated with FHR1, FHR2, FHR3, FHR4, FHR5, and C3, which encode components forming C3 or C5 convertases or regulators that govern the timing and site of C3 convertase activity [69]. However, a strict distinction between C3G and IC-MPGN is not always feasible. Patients with IC-MPGN also exhibit genetic and acquired disruptions in the alternative pathway. A retrospective study of 140 patients suffering from idiopathic IC-MPGN or C3G revealed a prevalence of genetic disorders not only in C3G, but also in IC-MPGN. The finding of mutations in the alternative C3 convertase components linked both diseases to alternative complement activation. Antibodies stabilizing the C3 convertase were also detectable in IC-MPGN, not only in C3G [70,71].

### 4.3. Lupus Nephritis (LN)

LN occurs in approximately 50–75% of cases of systemic lupus erythematosus [72]. It significantly determines the morbidity and mortality of the disease. The presence of autoantibodies, such as those against dsDNA, leads to the formation of immune complexes that can deposit in the kidneys. LN exhibits diverse clinical and morphological manifestations. Morphologically, there are six distinct types, with LN Type VI characterized by advanced sclerosis and, consequently, unresponsiveness to therapy. The focal LN (Type III) and diffuse LN (Type VI) often present a nephritic syndrome with a rapid decline in GFR. Type V LN morphologically shows a membranous pattern and frequently presents clinically as a nephrotic syndrome. Overall, LN demonstrates deposits of immunoglobulins and complement factors, often referred to as a “full-house pattern” [73]. Reduced serum levels of C3 and C4 serve as activity markers of LN [74]. Accordingly, the serum has elevated corresponding cleavage products such as iC3d, C4d, and C5b-9 [75,76]. The deposition of immunoglobulins and complement factors immediately suggests activation of the classical complement pathway in LN. This is supported by C4d deposits, which are frequently diffuse in Type III/IV LN but are subepithelial in Type V LN [77,78]. Thus, the location of complement activation and various dysregulations may lead to different types of LN [79]. Activation of the lectin pathway via increased MBL levels has also been described [80]. Another indicator of lectin pathway activation is the alteration of MASP1 and MASP2 in LN, which was observed in LN type III and IV compared to the membranous form [81]. Consequently, activation of the lectin pathway might contribute to the manifestation of different types of LN.

### 4.4. Focal Segmental Glomerulosclerosis (FSGS)

FSGS represents a histopathological description of morphological changes within glomeruli, predominantly characterized by segmental sclerosis. The challenge lies in recognizing that this histopathological pattern can emerge from many glomerulopathies. Often, FSGS represents the common endpoint of vastly different conditions mediated by adaptation (a mismatch between glomerular load and capacity), genetics, viral association (such as HIV), or medication-induced factors (e.g., lithium). In the primary form, akin to minimal change disease (MCD), there is an assumption of a circulating factor responsible for the disease [82]. Deposits of immunoglobulins like IgM and C3 are generally perceived as nonspecific. However, evidence is mounting regarding the involvement of the complement system in FSGS [83,84,85,86]. Serum studies in patients have revealed indications of complement activation, where complement cleavage products such as C3a, C5a, sC5b-9, C4a, and C4d were elevated [84,85,86]. The local regulation of complement becomes particularly intriguing. Angeletti et al. identified CD55/DAF as a crucial regulator of local complement activation in a mouse model of adriamycin-induced FSGS. A podocyte-specific knockout of DAF resulted in an increased C3b deposition in the glomeruli. The authors argue that C3a signaling in podocytes reduced nephrin expression [31]. Indeed, mutations in nephrin have been described in humans with FSGS [87]. Another study demonstrates a reduced DAF expression in FSGS patients, establishing a potential link to the human condition [88]. Even in the frequently described deposits of IgM, they might serve as effectors of the disease rather than nonspecific deposits. Trachtmann et al. describe IgM deposits in FSGS patients, correlating with deposits of complement products from the classical complement pathway. Furthermore, the authors speculate that IgM antibodies are not deposited nonspecifically but bind to glomerular antigens. It remains unclear whether antibody binding and complement activation contribute to disease onset or if these occur secondarily to glomerular damage. However, complement activation potentially contributes to disease progression.

### 4.5. Minimal Change Disease (MCD)

MCD is the most common cause of nephrotic syndrome in childhood. The disease was explicitly named due to the absence of morphological changes in light microscopy, including the absence of complement factors [89]. It is important to note that diagnosing MCD in childhood often relies not on biopsy, but on clinical criteria and response to cortisone. Biopsy for MCD is commonly performed in cases of atypical presentations or during adolescence or adulthood. This fact makes scientific analysis challenging. However, due to an immunologically mediated pathophysiology, interaction with the complement system is at least conceivable [90]. Nevertheless, current data regarding complement activation do not extend beyond measuring complement cleavage products in patients’ blood. Elevated levels of C5b-9 have been observed, while serum levels of C3 and C2 were reduced [88,91]. Even measurements of serum levels of C4a were inconclusive in different studies [92]. Therefore, a clear hypothesis regarding the involvement of complement in MCD cannot be conclusively generated.

## 5. Discussion

With the approval of Eculizumab, the therapy for aHUS has been revolutionized, shifting the focus toward the complement system in numerous glomerulonephritides [93]. Despite the successful MAC blockade in aHUS, complement-mediated kidney diseases remained elusive. Similar to aHUS, an overactivation of the alternative complement pathway leads to C3G. The inhibition of the terminal complement pathway is rarely effective in C3G; currently, no approved therapy exists [33,94]. It remains uncertain how to distinguish G3G from IC-MPGN. Despite the dominant immunoglobulin deposits in IC-MPGN, it frequently shares a histopathological pattern with C3G. Patients with IC-MPGN also exhibit alterations in the alternative complement pathway, which suggests that they might not be distinct diseases, and the immunoglobulin deposits in IC-MPGN could merely trigger complement activation [70,71]. Similarly, this scenario might apply to atypical postinfectious glomerulonephritis, often presenting as NS [95]. Typically, postinfectious glomerulonephritis follows a self-resolving course. However, in some cases, hematuria and proteinuria persist. Even in “atypical postinfectious glomerulonephritis”, underlying disturbances in the alternative pathway have been observed, suggesting that infection and immune complex deposition could serve as triggers for complement activation in this scenario. Overall, C3G is not primarily mediated by the terminal complement pathway but by mediators released from the proximal complement cascade [68]. Inhibition of the alternative complement pathway thus represents a potential therapeutic option. Currently, numerous drugs are in clinical trials, aiming to inhibit complement activation at various points (Table 1). However, these potential agents exhibit significant physiological differences. Iptacopan, an orally available small molecule inhibiting the activity of Factor B, has made the most progress. It aims to inhibit the alternative C3 convertase (C3bBb) and the alternative C5 convertase (C3bBbC3b), thereby inhibiting alternative complement activation at multiple points and reducing pro-inflammatory cleavage products. Iptacopan’s efficacy has been demonstrated by normalizing C3 levels in patient sera. Additionally, treatment resulted in a reduction of C3 deposits in the examined kidneys and was associated with a significant decrease in proteinuria [96]. An alternative approach involves the inhibition of Factor D, which is involved in the enzymatic cleavage of Factor B; however, this approach is not currently further pursued in C3G. Other investigated substances show less specificity toward the alternative complement pathway. Approaches include inhibiting the activity of the C3 convertase by binding to C3 or downregulating hepatic C3 production through siRNA. Avacopan, an inhibitor of the C5aR already approved for ANCA-associated vasculitis therapy, is currently undergoing clinical evaluation. With Avacopan, the goal is to reduce the pro-inflammatory effect of C5a while keeping the rest of the complement cascade intact [97].

In MN, there are varying perspectives on the primary complement activation. Seifert et al. investigated human biopsies associated with PLA2R1 and THSD7A-positive MN. As previously known, this study confirmed a dominant deposition of IgG4. However, IgG4 has a limited ability to activate complement. Yet, it was demonstrated that 100% of the 39 examined cases also exhibited another IgG isotype capable of complement activation (IgG1/IgG2/IgG3). In the case of MN, activation appears to occur primarily through immunoglobulins, specifically the classical pathway [62]. Conversely, other publications highlighting the central role of the alternative and lectin pathway exist [59,63]. Currently, when specific therapy is deemed necessary, the treatment of membranous nephropathy (MN) increasingly focuses on B-cell depletion. This approach aims to inhibit the production of autoantibodies [98]. Indirectly, this would also address activation through the classical pathway. Clinical trials investigating the specific inhibition of the classical and lectin pathways are in the early phases (Table 1). Utilizing the C3 inhibitor, pegcetacoplan, leads to the comprehensive inhibition of the complement system, thereby potentially achieving heightened efficacy. However, this approach comes with the trade-off of potential adverse events. To mitigate the latter, a more precise targeting of the complement system is appealing, with a consideration for specific modulation of the alternative pathway. The alternative complement pathway serves as an amplification mechanism for complement activation, operating independently or being triggered through the classical or lectin pathway [99].

Consequently, inhibiting the alternative pathway could be a therapeutic option for diseases primarily activated through the classical pathway. Lupus nephritis is characterized by deposits of antigen–antibody complexes (immune complexes), suggesting complement system activation through the classical pathway. Paradoxically, classical pathway complement factor mutations are considered risk factors for systemic lupus [100]. Classical complement activation appears to have a protective role by eliminating apoptotic cells. Therefore, inhibiting the classical complement pathway may not necessarily be beneficial. However, a potential approach could involve the specific complement blockade while preserving classical activation. Thus, along with inhibiting the alternative complement pathway, targeting specific complement blockade could also be considered in LN.

Diverse approaches to complement inhibition are emerging for all glomerular diseases. The current state of research does not allow for clear recommendations either in favor or against a specific inhibitor. Particularly in the case of IgA nephropathy (IgAN), uncertainties regarding complement inhibition as a therapeutic option are evident. IgAN is the most common primary glomerulonephritis globally [101]. It is an autoimmune disease characterized by the production of galactose-deficient IgA1 and antibodies against these IgA molecules. The mesangial deposition of these immune complexes leads to proliferation and glomerular infiltration [102]. The deposition of immune complexes can trigger complement system activation, with the exact mechanisms remaining poorly understood. Various complement activation pathways are discussed in the context of IgAN, focusing on the lectin and the terminal complement pathways. However, amplification via the alternative complement pathway is also considered, leading to ongoing clinical trials involving different complement inhibitors (Table 1) [103]. Promising results have been observed with the inhibition of the alternative complement pathway. Iptacopan, for instance, demonstrated a reduction in proteinuria compared to placebo in a Phase 2 study [104]. Data on C5 inhibition using Eculizumab were inconsistent, necessitating further study results. Surprisingly, despite a plausible pathophysiology for lectin pathway activation, the results of the investigation of Narsoplimab were disappointing. The expected reduction in proteinuria could not be achieved, leading to the termination of the study in 2023 [103]. The current understanding of the complement system is insufficient to predict therapeutic success, as indicated by the various studies and unexpected results of clinical studies.

For that, precise therapeutic planning is challenging because current diagnostics do not definitively determine complement activation at the time of diagnosis, nor can they identify the primary complement activation pathway (alternative, classical, or lectin). Urine would be an easily accessible biomaterial to measure complement activation. Indeed, there are data on complement products in the urine for the diseases discussed above [84,88,105]. However, these data are often derived from small case series, limiting normalization. Thus, it is conceivable that the levels of complement products in urine might not correlate with disease activity but with proteinuria or impaired reabsorption. A study from 2021 provided a large dataset of more than 16,000 patients. This publication normalized urinary complement products against proteinuria and GFR, and the analysis included a control cohort and numerous glomerular diseases. Analysis like this will help interpret data and identify potential biomarkers. For example, this work showed increased urinary excretion of complement factors after normalization to proteinuria in LN compared to systemic lupus. Even more intriguing was that some complement factors, such as Factor B, correlated less with GFR but were associated with specific entities (IgAN). As Factor B participates in the complement system’s alternative activation, this could reflect the primary activation (alternative pathway). Such data could be a diagnostic tool for complement activation and activity [92]. Efforts are also being made from kidney biopsies to quantify activity and identify the primary activation [62,106]. A proximity ligation assay identifies the primary complement activation pathway by distinguishing the different C3 convertase (C3bBb vs. C4bC2b) in renal biopsies. In the study by Person et al., a clear dominance of classical complement activation was evident in LN, while aHUS showed more activation of the alternative pathway [107]. In principle, with the expansion of such studies, an assignment to the primary complement activation in individual disease entities could be achieved. Additionally, the intensity of deposits might provide insight into the activity of the complement system at the time of examination. However, a definitive assignment to the primary activation of the complement system is not yet adequately possible.

Testing specific complement inhibition requires careful consideration in light of the myriad unresolved queries and uncertainties. Given the intricate nature of complement activation and the regulatory proteins involved, a thorough examination of activation patterns in both responders and non-responders from clinical studies becomes essential. This comprehensive assessment should encompass the analysis of plasma, kidney, and urine, potentially enabling the identification of distinct activation patterns. Such patterns could, in turn, facilitate the anticipation of treatment response in prospective scenarios. When contemplating the design of clinical trials, meticulous planning becomes paramount. Patient selection may necessitate a basis in their complement patterns, as discerned through a kidney biopsy. Moreover, the incorporation of surrogate endpoints is crucial, with a focus on measures of complement activation. This may encompass assessing blood and urinary levels of complement or employing staining techniques for complement products in repeated biopsies. Such considerations are particularly pertinent in the context of proof-of-concept studies.

In summary, the complement system plays a crucial role in various glomerular diseases. However, the exact mechanisms of its functioning are still not fully understood. The clinical manifestations of different diseases can be attributed to different patterns of complement dysfunction. A more detailed analysis can help to identify pathological subtypes, which can offer insights into the prognosis and activation of the disease. Therefore, a thorough understanding of the activation and control of the complement system within specific entities is necessary. This can facilitate personalized therapeutic decisions in the future.

## Figures and Tables

**Table 1:** Overview of current studies on complement system inhibition in various renal diseases.

Disease	Inhibitor	Pathway	Phase	NCT-Number
Primary membranous nephropathy	Iptacopan	Alternative (factor B)	II	NCT04154787
	Pelecopan	Alternative (factor D)	II	NCT05162066
	Narsoplimab	Lectin (MASP2)	II	NCT02682407
	Pegcetacoplan	Central (C3)	II	NCT03453619
	Gefurulimab	Terminal (C5)	I	NCT05314231
C3G	Iptacopan	Alternative (factor B)	II, III, OLE	NCT03832114, NCT04817618, NCT03955445
	NM8074	Alternative (factor Bb)	Ib	NCT05647811
	Pelecopan	Alternative (factor D)	II	NCT05162066
	Danicopan	Alternative (factor D)	IIa, IIb	NCT03124368, NCT03369236, NCT03459443, NCT03723512
	Narsoplimab	Lectin (MASP2)	II	NCT02682407
	TP10	Inhibition (scR1)	IIa	NCT02302755
	Pegcetacoplan	Central (C3)	II, III, OLE	NCT04572854, NCT03453619, NCT05067127, NCT05809531
	ARO-C3	Central (C3)	I/II	NCT05083364
	AMY-101	Central (C3)	I	NCT03316521
	KP104	Inhibition (CFH/C5)	II	NCT05517980
	Avacopan	Inflammation (C5aR)	II	NCT03301467
IC-MPGN	Iptacopan	Alternative (factor B)	III	NCT05755386
	Danicopan	Alternative (factor D)	IIa, IIb	NCT03124368, NCT03459443, NCT03723512, NCT03369236
	Pegcetacoplan	Central (C3)	II, III, OLE	NCT04572854, NCT05067127, NCT05809531
Lupus Nephritis	Iptacopan	Alternative (factor B)	II	NCT05268289
	Vemircopan	Alternative (factor D)	II	NCT05097989
	Narsoplimab	Lectin (MASP2)	II	NCT02682407
	ANX009	Classical (C1q)	I	NCT05780515
	Pegcetacoplan	Central (C3)	II	NCT03453619
	Gefurulimab	Terminal (C5)	I	NCT05314231
	Ravulizumab	Terminal (C5)	II	NCT04564339
IgA Nephropathy	IONIS-FB-LRx	Alternative (factor B)	II, III	NCT04014335, NCT05797610
	Iptacopan	Alternative (factor B)	II, III	NCT03373461, NCT04557462, NCT04578834
	Vemircopan	Alternative (factor D)	II	NCT05097989
	Pelecopan	Alternative (factor D)	II	NCT05162066
	Narsoplimab	Lectin (MASP2)	II, III	NCT02682407, NCT03608033
	ARO-C3	Central (C3)	I	NCT05083364
	KP104	Inhibition (CFH/C5)	II	NCT05517980
	Ravulizumab	Terminal (C5)	II	NCT04564339
	Avacopan	Inflammation (C5aR)	I	NCT06004947
